# A novel on design and implementation of hybrid MPPT controllers for solar PV systems under various partial shading conditions

**DOI:** 10.1038/s41598-023-49278-9

**Published:** 2024-01-18

**Authors:** Chakarajamula Hussaian Basha, Madhu Palati, C. Dhanamjayulu, S. M. Muyeen, Prashanth Venkatareddy

**Affiliations:** 1 NITTE Meenakshi Institute of Technology (Autonomous), Bengaluru, India; 2 BMS Institute of Technology and Management, Bengaluru, India; 3 School of Electrical Engineering, Vellore Institute of Technology, Vellore, 632014 India; 4 Qatar University, University Street, Doha, Qatar

**Keywords:** Energy science and technology, Engineering

## Abstract

At present, fossil fuel-based power generation systems are reducing drastically because of their less availability in nature. In addition, it produces hazardous gasses and high environmental pollution. So, in this work, the solar natural source is selected for generating the electricity. Due to the nonlinear behavior of PV, achieving maximum voltage from the Photovoltaic (PV) system is a more tough job. In this work, various hybrid optimization controllers are studied for tracing the working power point of the PV under different Partial Shading Conditions. The studied hybrid optimization MPPT methods are equated in terms of oscillations across MPP, output power extraction, settling time of the MPP, dependency on the PV modeling, operating duty value of the converter, error finding accuracy of MPPT, algorithm complexity, tracking speed, periodic tuning required, and the number of sensing parameters utilized. Based on the simulative comparison results, it has been observed that the modified Grey Wolf Optimization based ANFIS hybrid MPPT method provides good results when equated with the other power point tracking techniques. Here, the conventional converter helps increase the PV source voltage from one level to another level. The proposed system is investigated by using the MATLAB/Simulink tool.

## Introduction

Nowadays, the availability of nonrenewable energy sources is reducing rapidly. Also, nonrenewable energy sources give high atmospheric pollution. Due to the drawbacks of these sources, most industries are focusing on renewable energy sources^1^. From the literature review, the renewable sources are illustrated as tidal, wind, plus solar. Among all of the natural sources, PV is a more useful source since it has the features of the absence of noise generation, less maintained price, more reliability, high abundance, plus very low atmospheric pollution. In addition, it doesn’t need a high human source for operating the solar power plant^2^. The solar cells are manufactured by using various materials which are classified as cadmium telluride, silicon, gallium arsenide, crystalline silicon, and perovskite^3^. Among all of the materials, silicon is the most efficient material for PV cell design because its advantages are high energy efficiency, non-toxic material, cost-effectiveness, and good photoconductivity. The silicon solar cells are differentiated based on their efficiency which is illustrated as monocrystalline, amorphous, plus polycrystalline^4^. Based on the previously published articles, all of the PV manufacturers are utilizing the monocrystalline material because of its higher efficiency when compared to the other two semiconductor manufacturing technologies.

The single solar cell gives very little voltage which is nearly equal to 0.65–0.7 V which is not utilizable for high-power applications. So, the monocrystalline silicon cell joins in a series sequence to achieve the high output voltage. Similarly, the cells are arranged in a shunt manner to improve the current supply^5^. The PV working nature is equal to the basic diode operation. From the previously published articles, the cells are designed by applying the three various techniques which are defined as one diode PV cell, two diode PV cells, plus triple diode PV cells. In this article, a triple-diode PV technology is used for manufacturing the solar PV array. The features of 3-diode PV cells are high accuracy, more efficiency, plus a good fill factor. The solar cell operating nature is purely nonlinear. So, achieving more power from the PV array is quite complicated^6^. In solar PV standalone power distribution, there are four major key research areas involved which are MPPT design, PV cell selection, selection of suitable DC-DC converter for enhancing the PV supply voltage, and overall system performance enhancement^7^. The major problem of solar is the high per-unit power installation price which is compensated by utilizing the different MPPT concepts. The MPPT controllers are classified as conventional, artificial intelligence, soft computing, and swarm intelligence-based MPPT techniques^8^. The general power point finding methods are categorized as P&O, FOCV, Incremental Conductance (IC), FSCC, Incremental Resistance, ripple correlation, adaptive IC, and variable step value P&O controller.

The P&O method is used in^9^ for finding the operating point of the PV, and wind hybrid power supply system. In this P&O controller, the slope of the V-I curve is used for making the functioning point of PV near the actual working power point. The present I-V curve slope is compared with the past stored slope value^10^. The compared slope parameter consists of a negative value then it starts perturbing oppositely. Otherwise, the operation goes in a forward way. The features of this P&O method are simple in design, and good understanding. The demerits of the P&O method are less efficiency in the MPP tracking, suitability for constant irradiation, and temperature conditions of the PV systems. Also, this method is used where the precise MPP position is not needed. The disadvantages of this conventional controller are less applicable for dynamic irradiation conditions of the PV system^11^. The confines of the P&O are overcome by applying the IC concept^12^. The incremental conductance works with the help of the I-V curve. Here, the slope of the I-V curve conductance is monitored continuously until achieving the required MPP position. The features of IC are less dependency on solar panel design, good static response, and high-power extraction capability^13^. The major disadvantage is more implantation cost. The HC method is used in the solar-powered battery charging network. The hill climb controller gives fast tracking speed, less steady state settling time, and few oscillations of the operating point of PV^14^. The demerits of the HC concept are high dynamic oscillations of PV voltage, plus not as much of suitable for instant changes in the temperature values of solar light.

In^15^, the incremental resistance methodology is applied for improving the accuracy of MPP. In this method, the current density function is used to optimize the fluctuations of PV panel power. In the ripple correlation controller, the PV-fed boost converter gives ripple current and voltage which are fed to the MPPT block for obtaining the digital pulses to the power converter. But this method gives more power conduction losses^16^. So, the Kalman filter concept is used to minimize the oscillations of MPP by reducing the ripples of PV power. In^17^, the authors used the fractional voltage MPPT method for tracking the working point of the solar-powered traffic signal system. In this traffic solar power supply system, the high accuracy of power point tracing isn’t required. The merits of fractional voltage-based controllers are working simplicity, fast tracking speed of MPP, low time to convergence, and the required single sensor for sensing the PV voltage^18^. The demerits of this method are high switching losses at the time of measuring the PV open circuit voltage, and high complexity in obtaining the high efficiency of PV at various sunlight conditions^19^. Similar to the fractional voltage, the fractional current controller needed a single sensor for sensing the current parameter of PV. The slider controller-dependent MPPT block is interconnected in the middle part of the solar system to improve the entire system's working efficiency^20,21^. The slider-switching functions are determined from the nonlinear nature of the I-V curve. Based on the switching function of the slider, the working point of PV is adjusted until it reaches the actual MPP position.

However, the drawbacks of the above conventional controller are less efficient for the continuous changes in the atmospheric conditions of the solar PV system^22^. The dp/di, and dv/di slope values are calculated from the nonlinear features of the solar PV systems. After that, these values are fed to the power point tracking block for finding the functioning point of the PV. To limit the demerits of solar PV systems, most of the research scholars are working on the swarm, Chaos theory, soft computing, differential evaluation, metaheuristic, bio-inspired, and artificial intelligence related Maximum Power Point Tracking (MPPT) controllers^23^. The artificial intelligence controllers are reviewed in an article^24^ for optimizing the steady-state oscillations of the PV voltage and enhancing the load power of the DC-DC circuit from the partial shading condition of PV. In the ANN controller, any two combinations of solar panel parameters are selected for training the overall neural network. The training time depends on the number of layers in the network, and its learning has been done by utilizing the weight adjustment^25^. The available weights are kept on adjusted by using the backpropagation technique. Based on the backpropagation concept, the mean square error parameter is evaluated which is employed for identifying the MPP position. The major problem of neural networks is require complex data structures^26^. As a result, it takes more time duration for obtain a good tracing speed of MPP.

To limit the disadvantages of neural network controllers, a fuzzy logic concept is applied in^27^ to reduce the tracking time of the MPP. The fuzzy logic controller does not require any mathematical equitation solutions for optimizing the nonlinear problems. Also, it works for both precise and imprecise supply functions when compared to neural networks. Fuzzy system design may be a difficult task because of its appropriated membership utilization, and lack of mathematical foundations^28^. Also, the fuzzy controller may not give appropriate solutions for multiple numbers of rule base complex networks. So, the fuzzy disadvantages are limited by the combination of the Genetic Algorithm (GA), plus fuzzy. In this controller, the GA explores the more search space in a very effective manner and handles all types of fuzzy rules^29^. Here, the fitness function works to obtain the optimal solution for each candidate and provides the direction for their search processes. The GA identifies the suitable membership functions for the fuzzy by encoding the overall scheme, and representation^30^. The encoding accepts the crossover mutations and effective manipulation. The general encoding types are binary coding, plus linguistic encoding. The GA linguistic encoding may track any local optima instead of global optima. So, various strategies are considered for incorporating the diversity mechanisms which are elitism, and mutation constraints. In article^31^, the GA automatically optimizes the fuzzy rules. As a result, the PV modules lose the interpretability. However, the GA needed high computational complexity, accurate parameter tunning, premature convergence, less guarantee for tracking accurate MPP position, plus high sensitivity to the population initialization^32^.

According to the article^33^, the GA is integrated with the ANFIS controller for capturing the suitable MPP location of the induction motor-fed PV system. In this controller, the GA optimizes the parameters of solar PV, and ANFIS for increasing the inductive load voltage profile. Here, the population is converted into chromosomes which are initialized with various parameters like solar irradiations, PV array current, plus sunlight temperature^34^. The main work of GA is reducing the tracing time of the solar functioning point, and ANFIS optimizes the system's power loss by reducing the oscillations of the working point of the PV. The features of this hybrid methodology are high adaptability, and improved behavior, plus helps to achieve peak converter output voltage. Also, this method is applied for the fault diagnosis and fault detection of PV modules^35^. However, there are certain drawbacks to this method which are the curse of dimensionality, less interpretability for the larger number of inputs, plus more difficulty in finding the overall count of neurons^36^. In his work, there are different types of swarm optimization, and hybrid MPPT methodologies are studied for the enhancement of solar PV output voltage, and obtaining the suitable duty signal to the DC-DC converter circuit. From the previously available articles, the converter technologies are differentiated as rectifier-based converters, and non-rectifier-based converters^37^. In rectifier-based converters, the overall circuit is designed on a single chip. This rectifier included converters that offer high power quality, high voltage conversion, plus a high life span. The isolated converter topology is interfaced with the wind/PV system to supply equal power to the all-individual loads. The initial merits of this type of converter network are the separation of wind/PV output voltage, plus switches made to work with high efficiency^38^. Also, the feedback concept is utilized in the isolated converters for the system load power regulation, and ground loops are eliminated to reduce the implementation price of the overall power supply network of PV.

**Figure 1:**
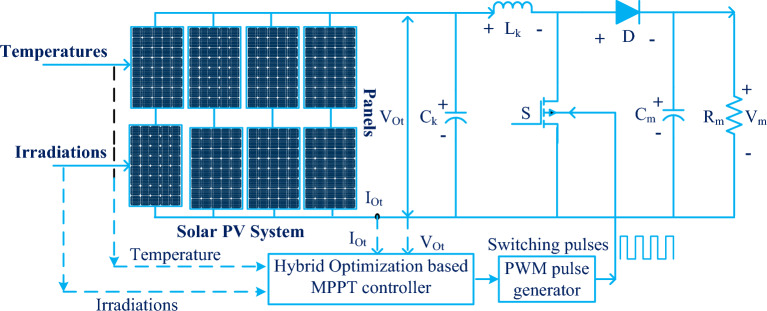
Proposed DC-DC converter circuit fed PV system with hybrid optimization MPPT controller.

The PV-fed electric drive system is operated by the use of a push–pull converter circuit for giving the rated power to the electric drive thereby reducing the converter voltage fluctuations^39^. The merits of a push–pull converter are the high capability of switching transient controlling, reduced power losses by complementary switching operation, plus more balanced operation with reduced consumer voltage ripples. The modified highly efficient forward DC-DC converter topology, and distributed MPPT methodology are used in mismatch phenomena of PV systems for maintaining the ripple-free load voltage^40^. This converter is investigated under various irradiation circumstances. The modified forward multiple inputs converter is used for wind as well as PV for effective distribution of load voltages. This power converter topology consists of an upper forward inductor, a common input inductor, and a lower forward inductor for smoothening of continuous distortions of wind/PV system voltages^41^. The upper, and lower forward inductors are operating simultaneously based on the atmospheric conditions. The features of the upper forward converter are cost-effectiveness, scaled up for high and medium power applications, high robustness, and good handling capability of a wide range of supply voltages. Also, this converter gives galvanic isolation between the source and the consumer^42^. As a result, the converter circuit noise generation is reduced. However, this circuit consists of high complexity in design, a more challenging task for controlling, a medium state of stability, and a high weight because of the additional transformer circuit^43^. Also, this circuit generates high electromagnetic interference due to the quick changes of switching frequencies, and voltage transitions. So, the PV voltage is improved by applying a non-isolated DC-DC circuit as shown in Fig. 1.

## Literature survey on solar MPPT controllers

All solar systems consist of nonlinear output performance. To limit the nonlinear nature of PV, there are multiple categories of power point tracing methodologies are selected for limiting and obtaining the optimal duty signal of the DC-DC converter circuit. In^44^, the authors state that they have reviewed nearly seventy-one articles on MPPT algorithms for uniform atmospheric circumstances of solar systems. From the retrospection of PV, the conventional algorithms are not useful for PV under multiple uniform irradiation values of sunlight. Also, they investigated the online, and hybrid algorithms by selecting five parameters which are PV module dependency, total number of iterations needed for adjusting the duty of inverters, design cost of the controller, error in MPP finding, plus efficiency of the entire utilized system^45^. Based on the investigation they concluded that the swarm intelligence methods give fast responses in all-weather situations. Similarly, the linear current control methodology is applied for the interleaved DC-DC circuit-fed PV modules to optimize the non-linear issue of PV by linearizing its V-I curve^46^. In this current control, the load line and MPP of PV are intersected at one position on P–V characteristics. Later, the differentiation of current concerning the power is made as zero for enhancing the peak power extraction of the solar system^47^. The capacitor droop methodology is applied to the single-stage interleaved DC-DC circuit for controlling the discontinuous power supply of PV. Here, the power equilibrium concept is utilized for identifying the suitable duty of the converter. As a result, the converter may supply good-quality voltage, and stable output power^48^. In addition, the PV supply is coupled with the AC system, and the inverter dc-link voltage, plus phase constraints are feedback to the pulse generator block for maintaining the constant grid power concerning the rapid changes of PV power^49^. Here, the V_link_ acts as a reference signal to the grid when the supply current rises excessively. The features of a power equilibrium controller are more power transferring capability, less size, plus acceptable implementation cost.

The adjustment of PV power curve slope and voltage curve slopes are fed to the pulse generator block for finding the MPP of sunlight-dependent PV system under quick time variant of irradiation conditions. The slope of the resistive load is maximum when the working point of PV is at the right-side corner of the V-I curve^50^. Otherwise, the slope value is zero when the load line intersects with the MPP. So, the duty adjustment is purely dependent on the sign of the PV voltage curve slope. In^51^, analytical technology is utilized for controlling the functioning temperature of the solar PV system in bad weather conditions. In this controller, the mean value theorem is involved in the adjustment of the open circuit voltage of the solar system. The advantages of the analytical technique are easy understanding, less size, plus fast system response^52^. For rooftop-installed solar systems, the gradient decent mathematical optimal concept helps to determine the P_MPP_ of PV under time-varying temperature values of the atmosphere^53^. The merits of the gradient decent technique are less dependency on PV cell manufacturing, few numbers of sensors applied for measuring PV power, plus current, and fast static response. However, these controllers are utilized for the static operation of PV to achieve more efficiency^54^.

In the modern smart grid power supply network, the neural controller is included in the P&O controller for improving the convergence speed of PV^55^. The wind/PV system output parameters are tuned by applying the neural network until identifying the global functioning point of the hybrid power network. The basic P&O works to reduce the tracing time of the solar MPP. The weighted point power point finding concept is applied to the solar P–V curve for optimizing the convergence time, and oscillations of MPP^56^. Here, there are various weighted points spread over the entire search space of PV curves for searching the suitable duty signal to the Z-source converter circuit fed FC/PV power system. The merits of a weighted point charge controller are more charging efficiency, a high-power conversion rate that works at high cold weather conditions, plus more robustness. The demerit of the weighted point controller is less suitable for rapid changes in the working temperature of the PV cell^57^.

The flower pollination and P&O combination controller is implemented for the interleaved high voltage conversion converter circuit-fed solar charger for maintaining the efficient state of discharge value. At the starting state, the P&O improves the convergence rate of the controller^64^. After that, the multiple numbers of flowers forecast the global MPP of PV in nonuniform weather conditions. However, this controller faces the computational issue that occurs due to the P&O working condition under continuous fluctuations of solar irradiation values^65^. The solar modules give very low supply voltages. Also, it works based on the load requirement. Based on the consumer loads availability, all the loads require high constant voltages without any distortions. So, the radial basis function power point identifying controller is integrated with the quadratic DC-DC converter circuit to enhance the working efficiency of solar PV^66^. From Table 1, the recently existing MPPT methods are analyzed for sunlight-powered PV systems under dynamic irradiation conditions.

**Table 1:** Analytical review of different types of MPPT methods for PV systems .

Name of authors	Published year	Selected parameters	MPPT method	Required signal	DC-DC converter	Major Evaluations
Vankadara et al.^58^	2022	Current, & voltage	MPA	Duty cycle	Boost converter	In this article, the Marine Predator Algorithm is used as an MPPT controller in the resistive load-fed PV system for limiting the shading behavior of PV modules. This controller is analyzed at four shading conditions of the PV network. Here, the authors tested the MPA controller experimentally by using the microcontroller and PV emulator. Based on the experimental analysis, the MPA works effectively with more accuracy
Muniyandi et al.^59^	2023	Irradiation, current, temperature	MSC with DE-AP&O	Duty cycle	Boost Converter	In this paper, the magic square concept is applied to PV cell configuration. Later, the differential evolutionary method integrated adaptive P&O methodology is utilized to optimize the issue of aforementioned. The magic square technology decreases the heating losses of the PV module, and DE-AP&O enhances the accuracy of MPP tracking at dynamic shading conditions
Abo-Khalil, Ahmed et al.^60^	2023	DC-link Voltage, PV current	SAA with P&O	Duty cycle	Buck-Boost	The authors combined the Simulative Annealing Algorithm (SAA) with the P&O to reduce the distortions of grid voltage. The PV cell may not work at MPP because of non-uniform sunlight temperature. As a result, the energy coming from the PV is not maximum. The simulative annealing concept improves the convergence rate of the P&O method. Here, the one-diode PV technology is used for a simple understanding of PV operation
Kumar et al.^61^	2023	PV current, power	SHC with QAC	Duty cycle	Buck & Boost	Based on environmental concerns, the demand for clean energy is increasing extensively. Especially, the popularity of PV utility is high when equated with the remaining sources. The PV power transfer efficiency depends on the loading factors, shading condition, and PV module functioning temperature. The identification of PV shading is a very crucial factor for improving the overall system life span. Here, the spotted hyena controller is combined with the quadrature approximation concept to improve the tracing speed of MPP
Hafeez, Muhammad Annas, et al. ^62^	2022	Load power, temperature	HHT with P&O	Duty cycle	CUK Converter	Here, the PV module is focused on mitigating the limitations of fossil fuels. There are multiple MPPs appear on the V-I curve of PV at quick changes in environmental conditions. To identify the global MPP, the authors introduced Harris Hawk’s technique with P&O for evaluating the peak current of PV under harmful weather conditions
Hu, Zhuxin, et al. ^63^	2022	Converter power, & dc-link voltage	Improved krill herd controller	Duty cycle	Bidirectional Converter	For economic profits, all of the consumers work on solar power to reduce the per unit cost of the central power system. The amount of power available from the PV is continuously dependent on environmental circumstances. Here, the fine-tuning of the fuzzy membership functions has been done with the help of the improved krill herd method

## Modeling and analysis of PV at shading conditions

**Figure 2:**
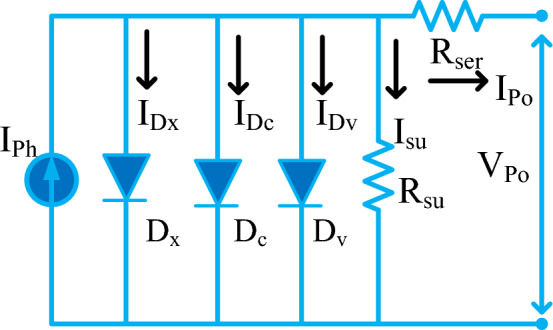
Solar cell with shunt resistance element^69^.

From the literature study, many more PV cell technologies exist in the market. The generally used PV module design technique is monocrystalline. This monocrystalline working efficiency is high when equated with other technologies such as poly, plus thin film^67^. The cells are interrelated to parallel to enhance the current profile of the system. Suppose, the PV system voltage is to be improved then the cells are interrelated by series. The cells are manufactured by utilizing various circuit topologies which are one-diode, dual-diode, plus triple-diode PV cells^68^. In this article, the triple diode methodology is selected for obtaining the precise nonlinear curves of PV. The working structure of the triple diode PV cell is shown in Fig. 2. From Fig. 2, the working condition of the solar cell is quite equal to the natural diode operation. Here, the sunlight falls on the PV array then the electrons move from the lower-level energy band to the higher-level energy band by the use of photon energy. The electricity generation of PV is dependent on the sunlight intensity, the cross-sectional area of the cell, and the sun's temperature.

Based on Fig. 2, the sunlight current is obtained by selecting Eq. (1). The advantages of the shunt element in the PV circuit are leakage current absorption, plus the improved performance of the overall PV network^70^.

1 $$ {\text{I}}_{{{\text{P0}}}}= {\text{ I}}_{{{\text{Ph}}}} -  {\text{ I}}_{{{\text{D}}_{{\text{x}}} }}  - {\text{I}}_{{{\text{D}}_{{\text{c}}} }} -{\text{ I}}_{{{\text{D}}_{{\text{v}}} }}  $$

2 $${{\text{I}}}_{{\text{P}}0}={{\text{I}}}_{{\text{Ph}}}-{{\text{i}}}_{{\text{re}}\_{\text{x}}}\left({{\text{e}}}^{\frac{{\text{q}}*({{\text{V}}}_{{\text{P}}0}+{{\text{I}}}_{{\text{P}}0}*{{\text{R}}}_{{\text{ser}}})}{{\upeta }_{{\text{x}}}*{\text{K}}*{\text{T}}}}-1\right)-{{\text{I}}}_{{\text{L}}}$$

3 $${{\text{I}}}_{{\text{L}}}={{\text{i}}}_{{\text{re}}\_{\text{c}}}\left({{\text{e}}}^{\frac{{\text{q}}({{\text{V}}}_{{\text{P}}0}+{{\text{I}}}_{{\text{P}}0}*{{\text{R}}}_{{\text{ser}}})}{{\upeta }_{{\text{c}}}*{\text{K}}*{\text{T}}}}-1\right)-{{\text{i}}}_{{\text{re}}\_{\text{v}}}\left({{\text{e}}}^{\frac{{\text{q}}({{\text{V}}}_{{\text{P}}0}+{{\text{I}}}_{{\text{P}}0}*{{\text{R}}}_{{\text{ser}}})}{{\upeta }_{{\text{v}}}*{\text{K}}*{\text{T}}}}-1\right)$$

where I_P0_, and V_P0_ are the solar module supply current, plus voltage respectively. The variables I_Ph_, plus I_Sh_ are the photon current, plus shunt current of the PV cell. Finally, the three diodes’ currents are indicated as I_Dx_, I_Dc_, plus I_Dv_. From Eq. (2), the charge recombination current increases then the photocurrent of the PV array is decreased gradually^71^. Also, the cell functioning temperature (T) rises from one state to another state then the power production from the supply is minimal, and it reduces the lifetime of the overall PV module. From Eqs. (5), and (6), the constraints ɳ_x_, ɳ_c,_ and ɳ_v_ are the ideality values for the three different diodes^72^. The ideality constant for the diode is more than the power loss of the PV array is high. In case, the shunt resistive element is included in the circuit then the sunlight current is derived as,

4 $${{\text{I}}}_{{\text{P}}0}={{\text{I}}}_{{\text{Ph}}}-{{\text{I}}}_{{\text{Dx}}}-{{\text{I}}}_{{\text{Dc}}}-{{\text{I}}}_{{\text{Dv}}}-{{\text{I}}}_{{\text{sh}}}$$

5 $${{\text{I}}}_{{\text{P}}0}={{\text{I}}}_{{\text{Ph}}}-{{\text{i}}}_{{\text{re}}\_{\text{x}}}\left({{\text{e}}}^{\frac{{\text{q}}({{\text{V}}}_{{\text{P}}0}+{{\text{I}}}_{{\text{P}}0}*{{\text{R}}}_{{\text{ser}}})}{{\upeta }_{{\text{x}}}*{\text{K}}*{\text{T}}}}-1\right)-{{\text{i}}}_{{\text{re}}\_{\text{c}}}\left({{\text{e}}}^{\frac{{\text{q}}({{\text{V}}}_{{\text{P}}0}+{{\text{I}}}_{{\text{P}}0}{{\text{R}}}_{{\text{ser}}})}{{\upeta }_{{\text{c}}}*{\text{K}}*{\text{T}}}}-1\right)-{{\text{I}}}_{{\text{K}}}$$

6 $${{\text{I}}}_{{\text{K}}}={{\text{i}}}_{{\text{re}}\_{\text{v}}}\left({{\text{e}}}^{\frac{{\text{q}}({{\text{V}}}_{{\text{P}}0}+{{\text{I}}}_{{\text{P}}0}*{{\text{R}}}_{{\text{ser}}})}{{\upeta }_{{\text{v}}}{\text{K}}*{\text{T}}}}-1\right)-\frac{{{\text{V}}}_{{\text{P}}0}+{{\text{I}}}_{{\text{P}}0}{{\text{R}}}_{{\text{ser}}}}{{{\text{R}}}_{{\text{sh}}}}$$

7 $${{\text{I}}}_{{\text{re}}\_{\text{x}}}={{\text{I}}}_{{\text{re}}\_{\text{c}}}={{\text{I}}}_{{\text{re}}\_{\text{v}}}={{\text{I}}}_{{\text{on}}} (\frac{{\text{T}}}{{{\text{T}}}_{{\text{n}}}}{)}^{3}{\mathrm{ e}}^{\frac{{\text{q}}*{\text{Eg}}}{{\text{nk}}}\left(\frac{1}{{{\text{T}}}_{{\text{n}}}}-\frac{1}{{\text{T}}}\right)}$$

8 $${{\text{I}}}_{{\text{on}}}={{\text{I}}}_{{\text{on}}\_{\text{x}}}={{\text{I}}}_{{\text{on}}\_{\text{c}}}={{\text{I}}}_{{\text{on}}\_{\text{v}}}=\frac{{{\text{I}}}_{{\text{SC}}\_{\text{n}}}}{{{\text{e}}}^{\left(\frac{{{\text{V}}}_{{\text{oc}}\_{\text{n}}}}{\upeta *{{\text{V}}}_{*{\text{Tn}}}}\right)}}$$

**Figure 3:**
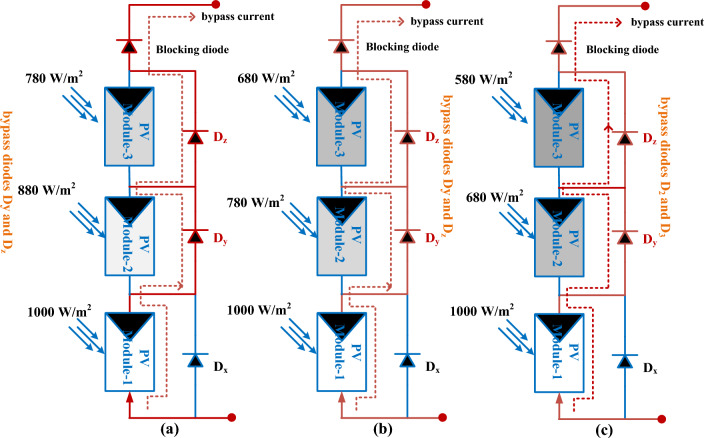
Solar modules at (**a**) PSC-1, (**b**). PSC-2, plus (**c**) PSC-3.

Most PV panels work economically under static environmental conditions^73^. But there are different effects on PV modules which are partial shading, plus bad weather conditions. These effects create an unbalanced power flow in the series of interlinked PV cells. The shading effect occurs on PV because of the building shadows, overcast weather, clouds, birds falling, overgrown trees falling, plus vegetation^74^. The neighbor houses and tall buildings may cast a shading on PV panels which is especially during some times of the day, and sunlight incident angle. Also, the overcast weather conditions reduce the direct sunlight absorption of the panels. Sometimes, the position of the sun varies continuously throughout the year because the earth tilts^75^. As a result, the sunlight inclination angle to the PV array is varied. Another major shading factor is the accumulation of soil on the solar module surfaces. Here, there are 3-shading patterns are assumed for the analysis, and investigation of solar systems as shown in Fig. 3a,b, and c. The design values of each solar module are explained in Table 2. From Fig. 3, it is identified that the shaded PV module-2 and module-3 are consuming the power of PV module-1. Due to these circumstances, the power production from supply may be affected. The diodes D_x_, D_y_, plus D_z_ are integrated across the PV cell and work to bypass the shaded panel current. The diodes are in a completely OFF state when the sunlight intensity falling on the PV is uniform. Otherwise, the diodes act in the conduction stage ^76^.

**Table 2:** Different shaded solar module design parameters.

S. No	Parameters	Values
1	Obtained voltage of PV under rapid changes of insolation conditions (V_MPP_)	30.18 V
2	Obtained current of PV under rapid changes of insolation conditions (I_MPP_)	8.3 amps
3	Obtained power of PV under rapid changes of insolation conditions (P_MPP_)	251 Watts
4	Evaluated PV open circuit voltage (V_oc_)	36.799 V
5	Evaluated PV short circuit current (I_sc_)	8.83 amps
6	Functioning temperature constant of voltage	−0.033002%/deg.c
7	Functioning temperature constant of current	0.061537%/deg.c
8	Diode operating constraints (ɳ_1_, ɳ_2_, ɳ_3_)	0.78, 0.83, and 0.961
9	Series linked resistance of cell (R_ser_)	0.2615 Ώ
10	Parallel linked resistance of cell (R_sh_)	251.311 Ώ
11	Lighting current of PV (I_ph_)	8.84536A
12	Saturation currents of diodes (I_re_x,v, w_)	1.45*e^-10^A
13	Utilized cells per module (N_s_)	60
14	The selected number of shading conditions	3

**Figure 4:**
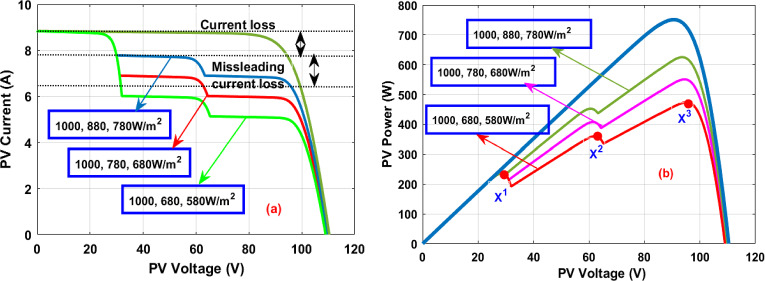
Solar PV generated, (**a**) I-V characteristics, plus (**b**) P–V characteristics.

The characteristics of solar modules under multiple insolation situations are given in Fig. 4a, b. Under constant irradiation conditions, the maximum available PV power (P_MPP_), current (I_MPP_), and voltages (V_MPP_) are 752W, 8.33A, plus 90.2 V respectively. At 1000, 880, plus 780W/m^2^, it is evaluated that there are three peak power points which are represented as X^1^, X^2^, plus X^3^. The parameters X^1^, plus X^2^ are the local functioning points of the solar module as shown in Fig. 4a. The required functioning point of PV is X^3^ which is operated by applying the MPPT controller. The available power, plus voltage from the PV under the initial PSC (1000, 880, plus 780W/m^2^) is 622W, plus 89.3 V, and it gives the PV supply current of 6.965A. The evaluated PV parameters like current, power, plus voltage under second rapid variations of irradiation conditions (1000, 780, plus 680W/m^2^) are 6.285A, 550W, plus 87.5 V respectively. Finally, at the third shading condition, the solar modules receive sunlight insulations of 1000, 680, plus 580W/m^2^ respectively. The obtained power of PV at the third shading condition is 475W, and its supplied voltage is 86.9 V. Here, from the PV curves, the industry power consumption from the PV is drastically reduced during night hours.

## Design of various MPPT controllers

PV cell efficiency is a more important factor for solar battery charging under cloudy conditions. The functioning efficiency of each cell is enhanced by maintaining the constant light intensity. As a result, the heating effect on the PV modules is balanced, and the extracted power from the supply PV module is more^77^. To maintain constant sunlight on PV modules, there are several power points identifying methods available in the literature. However, the demerits of those conventional strategies are moderate tracking speed, suitable only for uniform sunlight conditions, high power consumption at the time of perturbation, dependent on PV array manufacturing, a high number of sensors needed for sensing the PV parameters, plus more voltage ripples have existed in the converter generated power^78^. Also, these techniques may not apply to rapid changes in sunlight temperatures. In addition, the basic controllers give good efficiency when the sunlight incident angle same throughout the day. If the sunlight inclination angle is changing continuously concerning time, then the nature-inspired controllers are integrated with the sunlight system to maintain the output current of the cell within the limit. Here, the hybrid optimized MPPT controllers are studied under cloudy conditions of the solar PV system.

### Adaptive step value P&O MPPT controller

From the previously published articles, the P&O is the most generally utilized power point identifying controller for all the static insolation conditions of the hybrid solar power network^79^. This technique moves the functioning point of the PV from the one local point of the I-V curve to the global position. The implementation complexity of this methodology is easy. Also, it required only a few numbers of sensors for capturing the PV voltage and current data at various abnormal environmental conditions of the PV network. Mainly, this method may not depend on the modeling of the PV array. Here, the working efficiency of PV is improved by including the adjustable step constant value in the MPPT controller. When the working point of the PV is at the right side of the P–V curve then the adjustable constant value consists of + ve indication. Otherwise, the functioning point of PV may lie on the left side of the P–V characteristics^80^. The duty value of the converter is improved to move the functioning point of the PV near the actual MPP. The variation of DC-DC converter duty signal for the utilized PV-fed P&O MPPT controller is mentioned in Eq. (9). Based on EqS. (9), and (10), the variables D(a), plus P(a) are instant duty values and powers. The term ^‘^S^’^ is represented as the step value on the I-V curve of the PV array, and D(a-1), and P(a-1) are the past duty cycle and power values. Finally, the variables V(a), plus V(a-1) are instantaneous, and past voltages respectively.

9 $${\text{D}}({\text{a}})={\text{D}}({\text{a}}-1)+{\text{S}}*\left(\frac{{\text{P}}({\text{a}})-{\text{P}}({\text{a}}-1)}{{\text{V}}({\text{a}})-{\text{V}}({\text{a}}-1)}\right)$$

10 $${\text{D}}({\text{a}})={\text{D}}({\text{a}}-1)-{\text{S}}*\left(\frac{{\text{P}}({\text{a}})-{\text{P}}({\text{a}}-1)}{{\text{V}}({\text{a}})-{\text{V}}({\text{a}}-1)}\right)$$

### Successive approximation register-based MPPT controller

In this SAR controller, the Most Significant Bit is initialized with one digital value. This is one type of methodology used for the transformation of data from analog to digital form. Here, the sequential comparison has been done for the evaluation of each bit conversion result^81^. Suppose the sequential comparison gives a ^‘^1^’^ value then it identified that the most significant bit is not going to vary. If the comparison gives a ^‘^0^’^ indication then the MSB stored the zero value. From the literature, the identification of optimal power down on the I-V curve of the PV is a very critical task. The optimal power down stage is determined accurately by using the SAR controller then the overall power loss of the MPPT network is reduced. Otherwise, the SAR is stopped for finding the MPP position.

### Variable step-adaptive neuro-fuzzy inference system MPPT controller

From the literature survey, the neural networks are involved in the drawbacks of high computational expenses because of high training, and more capturing data of the solar systems. Also, it takes a long time duration for the development and requires highly talented candidates to gather the information^82^. The neural controller gives less efficiency of maximum power extraction from the PV because of the insufficient data collected by the neural network. Most recurrent neural networks work for time-dependent, plus sequential complex problems. Also, it works for stock market prediction, text writing, plus language translation. However, recurrent network training is very hard because of the gradient issue in the system. It especially suffers from the issue of vanishing gradients. The limitations of ANN are overcome by applying the fuzzy method. The accuracy of the fuzzy working is medium because of the imprecise data, plus membership function utilization. There is no particular way to sort out the issue of fuzzy membership consideration. In^83^, the researchers work on the fuzzy application in the hybrid PV/FC/wind microgrid power network. From the real-time implementation of the microgrid network, and its experimental analysis, the designed system gives more efficiency under abnormal conditions of the PV systems. However, it may not work effectively in shaded conditions of PV modules.

The ANFIS methodology is utilized in the article^84^ for reducing solar faults. ANFIS is a kind of neural network that works depending on the Takagi–Sugeno fuzzy inference model. It is developed from the principles of fuzzy logic, plus an artificial neural network. The major aim of ANFIS is to limit the disadvantages of fuzzy, and neural network controllers. The ANFIS inference is implemented from the various if–then rules. The rules of ANFIS approximate the PV function from a nonlinear way to a linear way. Due to that operation, the ANFIS systems are indicated as a universal estimator. In^85^, these ANFIS network optimal parameters are evaluated by applying the genetic algorithm. Also, this network is used in energy management systems for the smooth operation of PV-fed batteries. The variable step included ANFIS is developed in the PV/wind hybrid power network for achieving the required duty signal of the power converter. Here, the ANFIS controller memberships are mapped with the help of PV current, load voltage, plus supply voltage. The architecture involved the two supply variables that are G, and J, and its generated signal is M. The constraints V_1_, V_2_, W_1_, and W_2_ are the membership variables for the utilized variables.

11 $$\mathrm{if G is }{{\text{V}}}_{1} ,\mathrm{ plus J is }{{\text{W}}}_{1} ,\mathrm{ then K}={{\text{C}}}_{1}{\text{x}}+{{\text{H}}}_{1}{\text{y}}+{{\text{K}}}_{1}$$

12 $$\mathrm{if G is }{{\text{V}}}_{2},\mathrm{ plus J is }{{\text{W}}}_{2},\mathrm{ then K}={{\text{C}}}_{2}{\text{x}}+{{\text{H}}}_{2}{\text{y}}+{{\text{K}}}_{2}$$

where C_1_, H_1_, K_1_, C_2_, H_2_, and K_2_ are subsequent parameters of ANFIS. The selected adaptive neural network has five layers, and its layers are identified from the utilized membership values. The first layer nodes are two, and their output signals are evaluated as,

13 $${{\text{M}}}_{1,{\text{q}}}={\upmu }_{{\text{V}}1}\left({\text{x}}\right)+{\upmu }_{{\text{V}}2}\left({\text{x}}\right);\mathrm{ q}=\mathrm{1,2}$$

14 $${{\text{M}}}_{1,{\text{q}}}={\upmu }_{{\text{W}}1}\left({\text{y}}\right)+{\upmu }_{{\text{W}}2}\left({\text{y}}\right);\mathrm{ q}=\mathrm{1,2}$$

15 $${\upmu }_{{\text{q}}}\left({\text{x}}\right)=\frac{1}{1+{\left|\frac{{\text{X}}-{{\text{m}}}_{{\text{q}}}}{{{\text{n}}}_{{\text{k}}}}\right|}^{2{{\text{b}}}_{{\text{q}}}}}$$

From Eqs. (13) and (14), the variables $\upmu$ , ${{\text{M}}}_{1,{\text{q}}}$ are indicated as membership constants for the selected variables V, W. Here q, plus 1 are illustrated as several layers, and their related node. In^86^, the membership functions have various shapes which are Gaussian, plus trapezoidal. Also, the regularly used membership shapes are triangular, plus bell shapes. The variables n, m, plus b are weights of membership functions.

### Modified Grey Wolf MPPT controller for global MPP

The grey wolf controller is one of the general conventional methods, and this algorithm is developed from the natural behavior of wolves. The wolf's mimics are based on social leadership and its hunting principle. In this algorithm, there are four major parameters which are alpha, beta, delta, plus omega. The alpha (α) variable is illustrated as an optimal solution from the different possible solutions or leader wolf. Similarly, the beta (β), and delta (δ) constants are selected as the second, and third-best optimal solutions from the search space. The normal wolf solution is given by omega (ω). Here, the GWO is applied for the solar MPPT operation. However, the GWO MPPT method does not provide the best optimal duty for the DC-DC power converter because of the larger population size, highly selected search space, plus required high number of wolf solutions. The current position of the wolf is adjusted by utilizing the linear tunning vector ($\overline{{\text{S}} })$. Due to the vector ($\overline{{\text{S}} })$ linear tunning, the convergence rate of GWO is very low. As a result, the exploration, plus exploitation process of the grey wolf controller is continuously imbalanced. So, the modified grey wolf concept is introduced in the MPPT block for balancing the population as well as searching space for the wolves. In this modified grey wolf mechanism, the position of a particle is increased by the application of swarm optimization. As a result, the wolf's exploration, and convergence ratio are optimized extensively^87^.

**Figure 5:**
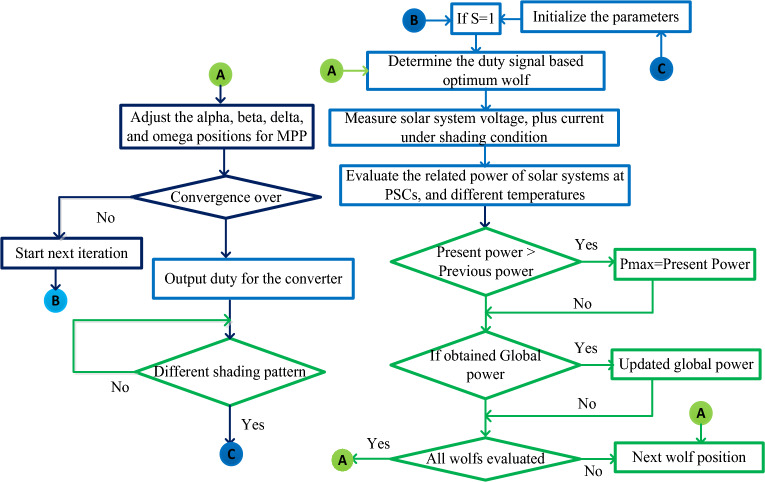
Modified grey wolf-based power point identifying controller for solar PSCs.

The updated position of the wolf has been determined by applying Eq. (16). Here, each particle's speed, and position are continuously updated until achieving a good exploration process, and optimal accuracy of MPP tracking. The solar MPP accuracy depends on the new position, plus the past position of the wolfs. Also, from the wolf's optimal exploitation process, the settling time of solar MPP is reduced. The features of this modified grey wolf methodology are highly efficient operation under cloudy circumstances of PV modules, less PV heating losses, optimal iterations utilization of wolf, less hardware implementation cost, plus less power electronic diode losses. From Eq. (16), the variable $\overline{{W }_{MGWC}}(x+1)$ is selected as the present updated random position of the wolf, plus ^‘^x^’^ is illustrated as the iteration number. The term ${\overrightarrow{S}}^{1}$ is the last selected best particle position among the different random positions. The constraints ${C}_{1}\in \left(\mathrm{0,1}\right), \& {C}_{2}\in \left(\mathrm{0,1}\right)$ are the coefficients of wolves which are applied for the regulation of the exploitation process.

16 $$\overline{{{\text{W}} }_{{\text{MGWC}}}}\left({\text{x}}+1\right)={{\text{C}}}_{1}*\overline{{{\text{W}} }_{{\text{GWC}}}}\left({\text{x}}+1\right)+{{\text{C}}}_{2}*({\overline{{\text{X}}} }^{1}-\overline{{\text{X}} })$$

17 $$\overline{{\text{S}} }\left({\text{t}}\right)={{\text{S}}}_{{\text{intial}}}-\left({{\text{S}}}_{{\text{intial}}}-{{\text{S}}}_{{\text{final}}}\right)*\left(\frac{{{\text{Max}}}_{{\text{it}}}-{\text{x}}}{{\text{Maxit}}}\right)$$

where S_intial_, and S_final_ are the initial, and final linear tuning parameters which can be varied based on the total number of iterations used in the GWO. Finally, Max_it_ is indicated as the final iteration of the grey wolf controller. The detailed initialization of the grey wolf and its nature of workflow is defended in Fig. 5.

### Hybrid adaptive P&O-PSO power point identifying controller

In the article^88^, the authors worked out the different hybrid controllers for sunlight-based PV systems to enhance the voltage stability of the microgrid system. Here, in the P&O controller, the different step value is applied for running the functioning point of the PV array almost near the required MPP. Due to the different step values, the P&O controller is useful for dynamic operating temperature conditions of the sunlight PV systems. This general conventional controller design is easy and requires very few sensors. So, the maintenance cost of the P&O controller is much less when equal to the differential evaluation controllers. The major complication of this controller is power distortions of the sunlight system under quick variations of irradiation circumstances. Also, this controller may not be capable of handling the multiple MPPs of the solar system.

**Figure 6:**
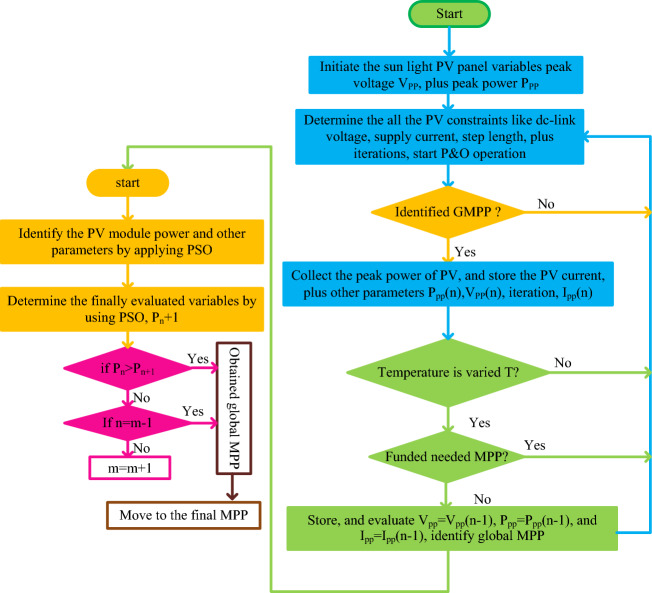
Modified grey wolf-based power point identifying controller for solar PSCs.

As of the current solar power supply scenario, the Particle Swarm Optimization (PSO) concept is utilized in many standalone, and microgrid power supply systems for handling the energy at peak load conditions. In the microgrid system, the PSO controller supplies the pulses to the bidirectional converter for efficient battery charging. The depth of charge, plus the depth of discharge of the battery are monitored by utilizing the artificial intelligence-based energy management system. However, the search space of the P–V curve of the solar system is improved then the working iterations of the PSO are increased. So, the solar PV MPP tracking time is increased. Also, it may not give more accuracy of PV voltage under dynamic as well as cloudy conditions of the solar systems. In^89^, the researchers interfaced the P&O controller with the PSO block to improve the PV supply power. In this hybrid method, at the start, the general P&O concept is introduced for optimizing the MPP tracking speed under multiple sunlight temperature circumstances. At the final state of the solar MPP tracking, the P&O jumps to the PSO controller to minimize the fluctuations of the resistive load voltage. In the PSO searching mechanism, the population velocity, plus step positions are varied by selecting Eqs. (18), and (19). This hybrid MPPT technique workflow is given in Fig. 6.

18 $${{\text{J}}}^{{\text{m}}+1}={{\text{WJ}}}_{{\text{i}}}^{m}+{\delta }_{a}{\upmu }_{{\text{a}}}({{\text{P}}}_{{\text{best}}}-{n}_{{\text{i}}}^{{\text{m}}})+{\delta }_{b}{\upmu }_{{\text{b}}}({{\text{G}}}_{{\text{best}}}-{n}_{{\text{i}}}^{{\text{m}}})$$

19 $${{\text{n}}}^{{\text{m}}+1}={{\text{n}}}_{i}^{{\text{m}}}+{{\text{J}}}_{{\text{i}}}^{{\text{m}}}$$

where J, W, plus P_best_ are the population velocity, weight of each particle, plus local best PV output power. The terms m, and i are particle numbers and iterations. Finally, the terms n, ${\delta }_{a}$ and ${\delta }_{b}$ are the particle position, and empirical coefficients.

### Modified GWO-ANFIS power point identifying controller

From the current article publication status, the basic conventional methodologies are not useful for the dynamic working conditions of solar systems because their drawbacks are more convergence time, less speed of MPP tracking, more distortions in supply power, required high-cost sensors, more size, and cost. In article^90^, the artificial NN is applied for the PV parameters identification in order to interconnect the renewable PV source with the grid connection. Solar power charges the battery when the excess supply is available from the system. Otherwise, it gives power to the central grid. For the grid integration of the solar system, the PV module voltage exactly matches with the grid voltage which can be done by utilizing the NN controller. The merits of NN’s when equated with the fractional voltage, and current methods are less complexity in implementation, less catchment area for the installation, and fast response. Here, the ANN is trained with open circuit voltage, multiple irradiations, different temperatures, plus various ideality factors. The entire data of NN is trained by utilizing the Levenberg–Marquardt concept^91^.

**Figure 7:**
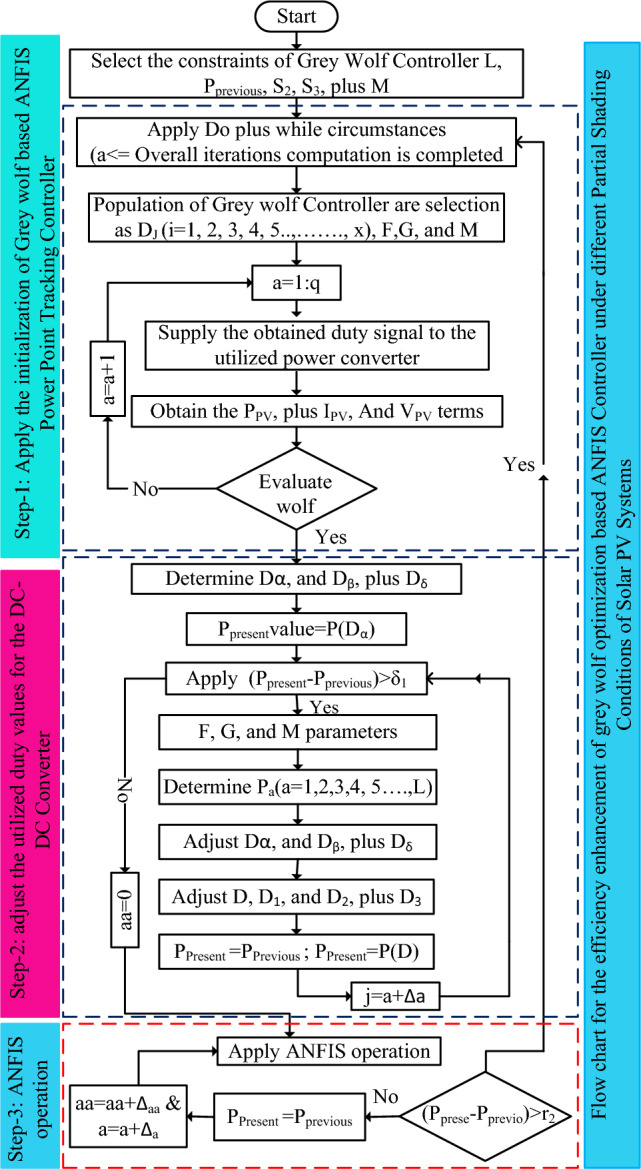
Proposed hybrid GWO-ANFIS power point identifying controller for PSCs of PV systems.

The drawbacks of conventional NN are less precise in MPP finding, high complexity in the network when a greater number of layers are selected, plus less nonlinearity problem-solving capability. The fuzzy logic concept is applied to the hybrid grid-connected renewable energy systems for the proper allocation of distribution loads. The fuzzy handles the inaccurate input variables, and it does not use any mathematical models for the implementation of the MPPT controller. Fuzzy can handle any nonlinear issue with high efficiency for obtaining the peak power from the PV systems. However, fuzzy networks are not used for the PSCs of PV systems. Also, in some cloudy cases of solar PV systems, the accuracy of the fuzzy is very less because of inaccurate supply data. In addition, it does not have any unique systematic approach to solving complex issues. In this article, the combination of enhanced grey wolf controller, plus ANFIS is utilized for identifying the global MPP of PV at quick variation of irradiation conditions. The ANFIS is developed from the merits of fuzzy, and neural network methods. In this ANFIS controller, the membership functions selection has been done by the use of the grey wolf controller. The hybrid grey wolf-fed ANFIS controller's working behavior is given in Fig. 7. From Fig. 7, the grey wolf controller starts working at local peak power points of the solar PV systems. After the identification of all local MPPs, the grey wolf controller signals are fed to the ANFIS block to reduce the oscillations of global MPPs. The combination of ANFIS, and GWO controller helps the solar PV systems for running under abnormal environmental conditions. From Fig. 7, the error values obtained from the grey wolf block are fed to the ANFIS controller. Similarly, the collected error signals from the ANFIS are fed back to the GWO as given in Eq. (21).

20 $$\left|{{\text{P}}}_{{\text{present}}}-{{\text{P}}}_{{\text{previous}}}\right|\le {\updelta }_{1}$$

21 $$\left|{{\text{P}}}_{{\text{present}}}-{{\text{P}}}_{{\text{previous}}}\right|\ge {\updelta }_{2}$$

where P_previosu_, and P_present_ are the past determined PV array power, plus instant determined PV array power. The parameter ${\updelta }_{1}$ helps supply the error signal to the ANFIS block. The selected utility constant ${\updelta }_{1}$ for this hybrid power point identifying method is 0.06. Based on this selected utility value, the ANFIS-obtained output signal is fed back to the GWO block. Similarly, the parameter ${\updelta }_{2}$ applied to the grey wolf block for accepting the ANFIS-generated error signals.

## Operation and analysis of power DC-DC converter

The PV power supply per unit price is high which can be decreased by the use of different power electronics converters^92^. The isolated converter circuits are flyback, push–pull, plus forward converters. In the flyback converter structure, there are three stages of operations included which are two voltage-controlled modes, plus one current supply control strategy. Most solar PV module manufacturers utilize the current mode operation for the efficient operation of the converter circuit, and this mode gives high voltage stability as well as constant load power. This flyback topology is applied in renewable grid integration systems for the conversion of AC to DC power by utilizing the galvanic isolation concept at the input side of the converter circuit. The inductors of the flyback circuit stored the electricity under the switch-blocking condition^93^. In this converter, the load regulation happens due to the sudden variations of switching states. The complexity of this flyback circuit is high because of its integration with the snubber circuit for switching protection. Also, requires a high current-rated capacitor for high load voltage applications, and this converter efficiency is very poor because of its pulsating source current. In^94^, the researchers used the forward model power converter for the solar power-fed water supply application. The reliability of this forward converter circuit is high because of its simple structure when equated to the bridge model power converter.

Most of the forward circuit topologies are placed near the source to prove highly efficient voltage transmission from the supply. The forward converter circuit voltage is adjusted with the help of inductive turns of it. This converter circuit does not have any ground connection and it used the translator network for proving the efficient ground to the forward converter. As a result, the entire forward circuit cost, plus size is increased. In recent years, the dual switch forward converter has been applied to the FC/PV-based microgrid networks for providing continuous power to customers. Due to these dual switches, the converter power supply stress is optimized on the switches, and it eliminates the transformer winding reset. The push–pull converter circuit is applied for many industrial high-voltage applications^95^. This type of converter circuit is used in supercapacitor banks, industrial drives, and high-voltage solar inverter applications. However, the above-isolated converter circuits needed more cost for design and high complexity in maintenance.

**Figure 8:**
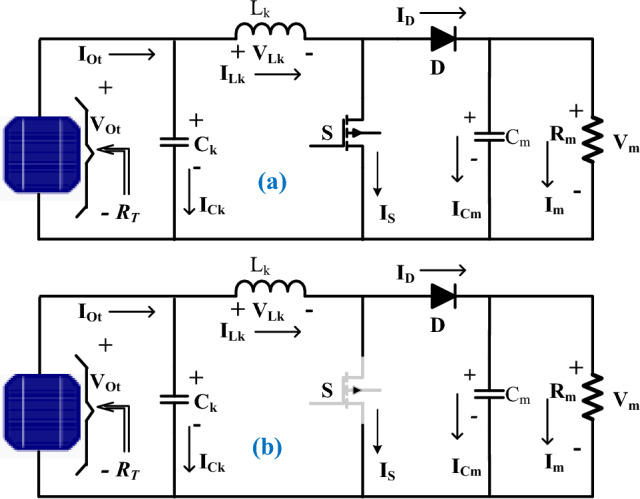
Utilized power DC-DC converter circuit, (**a**) ON state, and (**b**) OFF state.

So, from the present published works, the major power converters applied for the solar power supply systems are non-isolated DC-DC power converters because their design complexity is much less, and requires a very small size, plus less space for the installation. Here, in this work, the obtained PV voltage is supplied to the power DC-DC converter for enhancing the PV source voltage to meet future microgrid voltages. The working operation of the converter is clearly stated in Fig. 8a, b. Based on Fig. 8, the inductor voltage, plus current charging, plus discharge balancing are derived in Eqs. (22), and (23). From Eq. (12), the parameters V_Ot_, R_T,_ and I_Ot_ are identified as the supply voltage of PV, PV equivalent resistor, plus PV current^96^. The converter's working time duration is indicated as ^‘^T^’^, and its duty cycle is defended as ^‘^D^’^.

22 $${{\text{DT}}}_{{\text{k}}}*{{\text{V}}}_{{\text{Ot}}}+(\left({{\text{V}}}_{{\text{Ot}}}-{{\text{V}}}_{{\text{m}}}\right)*\left(1-{\text{D}}\right){{\text{T}}}_{{\text{k}}}=0$$

23 $$ - {\text{I}}_{{\text{m}}} {\text{*DT}}_{{\text{k}}} + (\left( {{\text{I}}_{{{\text{Ot}}}} - {\text{I}}_{{\text{m}}} } \right){*}\left( {1 - {\text{D}}} \right){\text{T}}_{{\text{k}}} = 0 $$

From Eqs. (22) and (23), the evaluated voltage, plus the current of the converter circuit are mentioned in Eqs. (24), and (25). Also, the converter inductor and capacitor design formulas are given in Eqs. (28) and (29).

24 $${{\text{V}}}_{{\text{m}}}={{\text{V}}}_{{\text{Ot}}}/ (1-{\text{D}}),\mathrm{plus }{{\text{i}}}_{{\text{m}}}={{\text{i}}}_{{\text{Ot}}}\left(1-{\text{D}}\right)$$

25 $$\frac{{{\text{V}}}_{{\text{m}}}}{{{\text{I}}}_{{\text{m}}}}=\frac{{{\text{V}}}_{{\text{T}}}}{{{\text{I}}}_{{\text{T}}}}\left(\frac{1}{{(1-{\text{D}})}^{2}}\right)$$

26 $$\frac{{{\text{V}}}_{{\text{m}}}}{{{\text{I}}}_{{\text{m}}}}={{\text{R}}}_{{\text{m}}}; \frac{{{\text{V}}}_{{\text{Ot}}}}{{{\text{I}}}_{{\text{Ot}}}}={{\text{R}}}_{{\text{T}}}$$

27 $${{\text{R}}}_{{\text{T}}}={{\text{R}}}_{{\text{m}}}*\left({(1-{\text{D}})}^{2}\right)$$

28 $${{\text{L}}}_{{\text{k}}}=\frac{{{\text{V}}}_{{\text{m}}}*{\text{D}}}{\Delta {{\text{I}}}_{{\text{Lk}}}*{{\text{f}}}_{{\text{s}}}}$$

29 $${{\text{C}}}_{{\text{k}}}=\frac{{{\text{I}}}_{{\text{Lk}}}{\text{D}}*\mathrm{\Delta t}}{\Delta {{\text{V}}}_{{\text{PV}}}}$$

## Analysis of simulation results

The solar system-fed converter circuit is implemented by utilizing the MATLAB Simulink tool. The solar system installation and its working is entirely depending on the sunlight inclination angle. Also, at quick changes in sunlight conditions, the solar PV modules give very little power. So, the converter is connected to the PV output to improve the power supply capacity of the solar system. The value of the source side capacitor (C_K_) is equal to 185µF, and it works for filtering the PV power ripples. Also, this capacitor is useful for the protection of the switches at rapid variations of supply voltages. The utilized supply inductor (L_k_) is selected as 18mH. This inductor charges the supply voltage of PV under the MOSFET forward bias condition. After that the switch is in a block state then the inductor supplies the voltage to the load. Also, this inductor smoothening the supply voltage. Here, the MOSFET is used because its properties are less gate-to-source voltage required for going to conduct, operates high efficiency at low rated voltages, consists of high source resistance to limit the gate current, quick working response, plus less possibility of damage. The capacitor (C_m_) is selected for the load voltage maintenance, and it is selected as 200µF. Also, this capacitor stores the electrostatic energy of the PV panel for rapid changes in sunlight temperature values. The R_m_ load resistor is connected to the parallel to the capacitor C_m_ as given in Fig. 8.

### Investigation of the solar PV panel at 1000W/m^2^, 880W/m^2^, and 780W/m^2^

At cloudy environmental conditions, the solar systems supply rapid changes of power generation for all-local consumers without any MPPT block. For removing the fluctuations of PV power, the power point identifying controller is connected to the converter source side. Here, there are multiple MPPT methodologies are applied to make the PV system run at peak power point position. The differentiate step value-P&O, adaptive SAR, plus VS with ANFIS controllers gives the PV panel voltage, and powers of 86.13 V, 585.68W, 87.92 V, 599.61W, 88.62 V, and 608.81W respectively. The adaptive SAR block, DSV-P&O, and VS with ANFIS controllers generated duty signals to the converter under initial cloudy conditions of the PV system are 0.63, 0.78, plus 0.52. Here, the ADSV with P&O block is taking a higher converter duty cycle ratio for obtaining the peak power. So, the entire PV panel power distribution losses are more for all local loads. The power, plus current of the converter by utilizing P&O-PSO, Modified GWO, plus multiple steps based ANFIS controllers are 601.14W, 4.343A, 594.60W, 4.3649A, 591.58W, plus 4.382A. Similarly, the load current, plus load powers of the converter by integrating the MGWO-ANFIS at PSC-1 are 4.259A, plus 606.15W.

**Figure 9:**
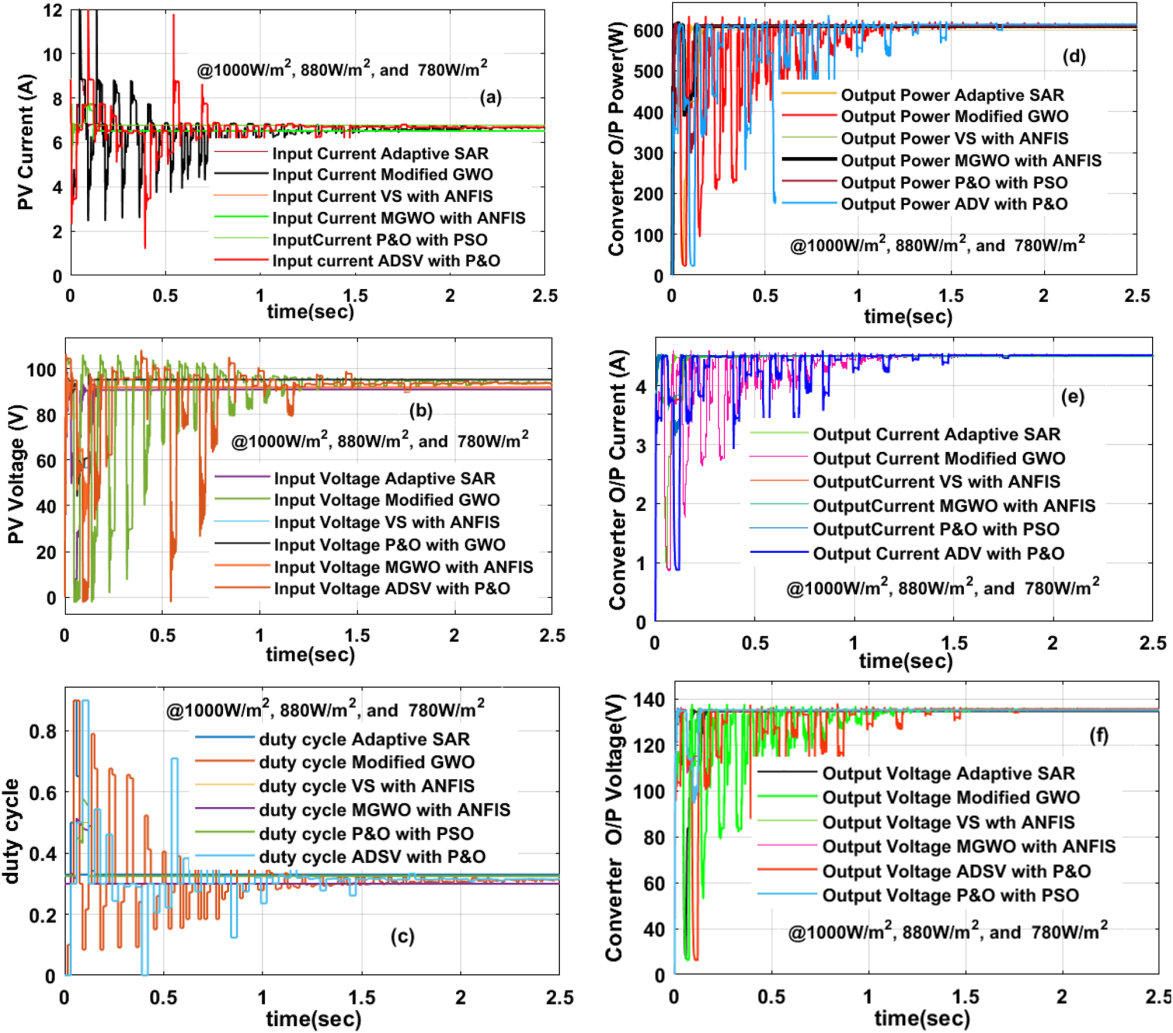
Under 1000W/m^2^, 880W/m^2^, and 780W/m^2^, (**a**) PV panel current, (**b**) PV panel voltage, (**c**) the duty cycle of the converter, (**d**) Power of converter, (**e**) the current of the converter, plus (**f**) voltage of converter.

The obtained PV modules settling time of the current under multiple step irradiations by using adaptive SAR is 0.13 s as given in Fig. 9a. The dynamic response of the PV voltage by integrating the MGWO-ANFIS MPPT block is very good when equated with the modified GWO as explained in Fig. 9b. Also, this controller takes very little time for the convergence to find out the needed MPP of the PV. The duty cycle of the converter is not constant until the PV panel reaches the MPP position, and it is mentioned in Fig. 9c. The aim of the converter is the enhancement of power efficiency. By the use of adaptive SAR, VS with ANFIS, plus MGWO-ANFIS, the obtained converter power efficiency is 97%, 97.17%, plus 98.20% respectively. The evaluated power, plus current of the converter waveforms are given in Fig. 9d, e. The generated converter voltage by applying the ADSV with P&O, plus P&O-PSO is 134 V, plus 138.41 V which is illustrated in Fig. 9(f).

### ***Investigation of the solar PV panel at 1000W/m***^***2***^***, 780W/m***^***2***^***, and 680W/m***^***2***^

**Figure 10:**
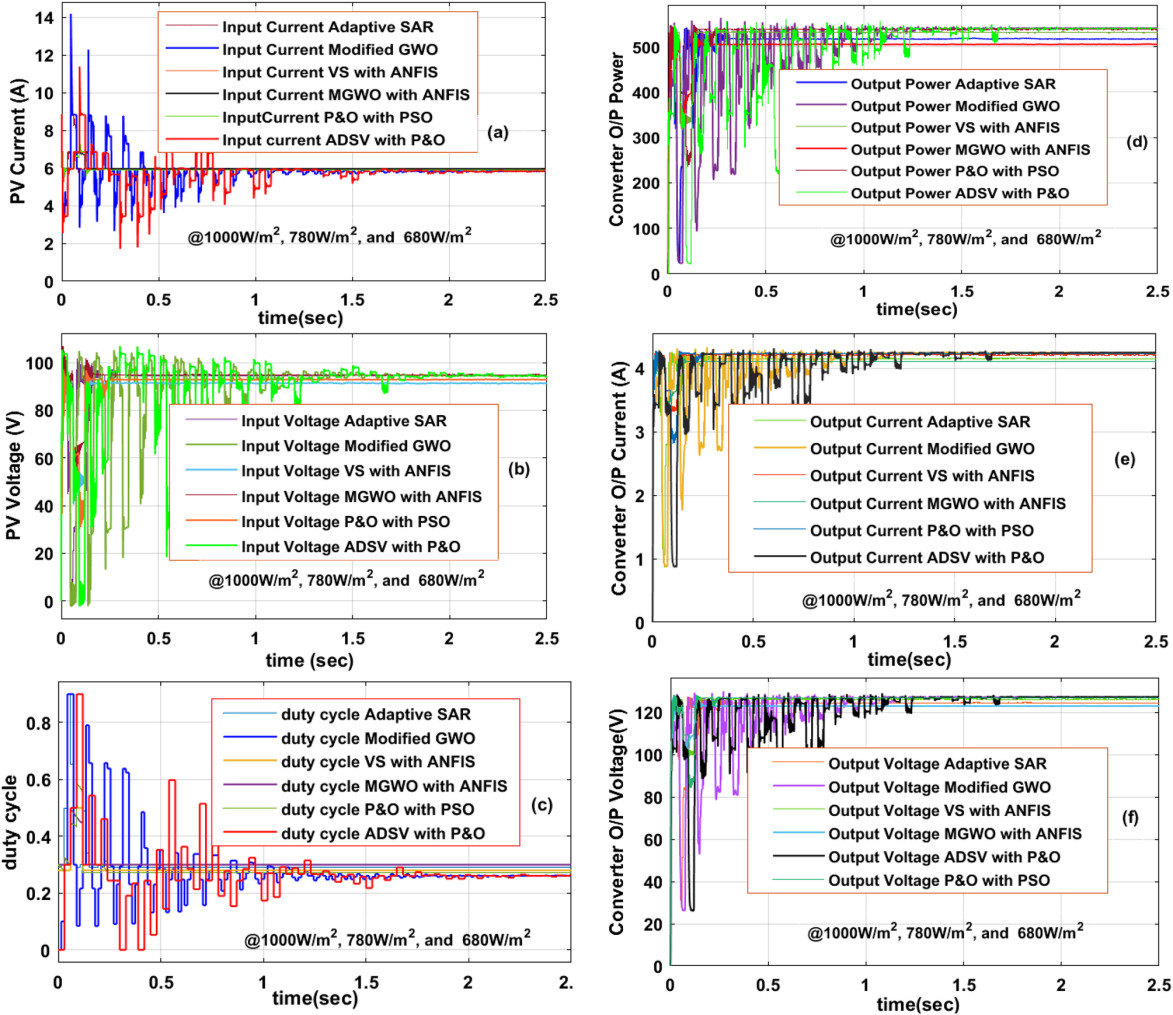
Under 1000W/m^2^, 780W/m^2^, and 680W/m^2^, (**a**) PV panel current, (**b**) PV panel voltage, (**c**) the duty cycle of the converter, (**d**) Power of converter, (**e**) the current of the converter, plus (**f**) voltage of converter.

From Sect. "Literature survey on solar MPPT controllers", the PV panel current is generated depending on the sunlight irradiance. Here, the irradiation fell from the higher level to the lower level. Due to that the peak extracted power is reduced. At PSC-2, the evaluated PV currents, plus voltages by merging the ADSV with P&O, Adaptive SAR, VS with ANFIS, plus modified GWO blocks are 6.17A, 86 V, 6.18A, 87.18 V, 6.2A, 87.22 V, 6.212A, plus 87.39 V respectively. The overall MPPT controller PV panel current, plus voltages are illustrated in Fig. 10a, b. From Fig. 10a, the stabilizing time of the PV current by merging the MGWO-ANFIS, modified GWO, VS with ANFIS, plus adaptive SAR is 0.027 s, 0.098 s, 0.188 s, plus 0.21 s respectively. The PV panel's proper power supply to the grid is mainly focusing on the voltage balancing between the source and local loads. Based on the duty cycle control of the converter, the PV voltage, and grid voltages are run at the same phase. From Fig. 10c, the determined duty cycle of the converter by merging the SAR, plus P&O is 0.81, plus 0.73. So, the conventional controller needed a high duty value to reach the peak power position of the solar system. Due to these circumstances, the converter gets heating losses. The converter supplied different voltages, plus various currents by applying the adaptive SAR block, DSV-P&O, and VS with ANFIS controllers are 127.59 V, 4.086A, 127.11 V, 4.077A, 127.66 V, plus 4.1A. Here, the ANFIS-fed PV voltage is more and gives moderate complexity in design. The complete converter voltage waveforms, plus current waveforms are mentioned in Fig. 10d, e. Finally, the load-generated power waveforms by applying all the MPPT techniques are shown in Fig. 10f.

### ***Investigation of the solar PV panel at 1000W/m***^***2***^***, 680W/m***^***2***^***, and 580W/m***^***2***^

**Figure 11:**
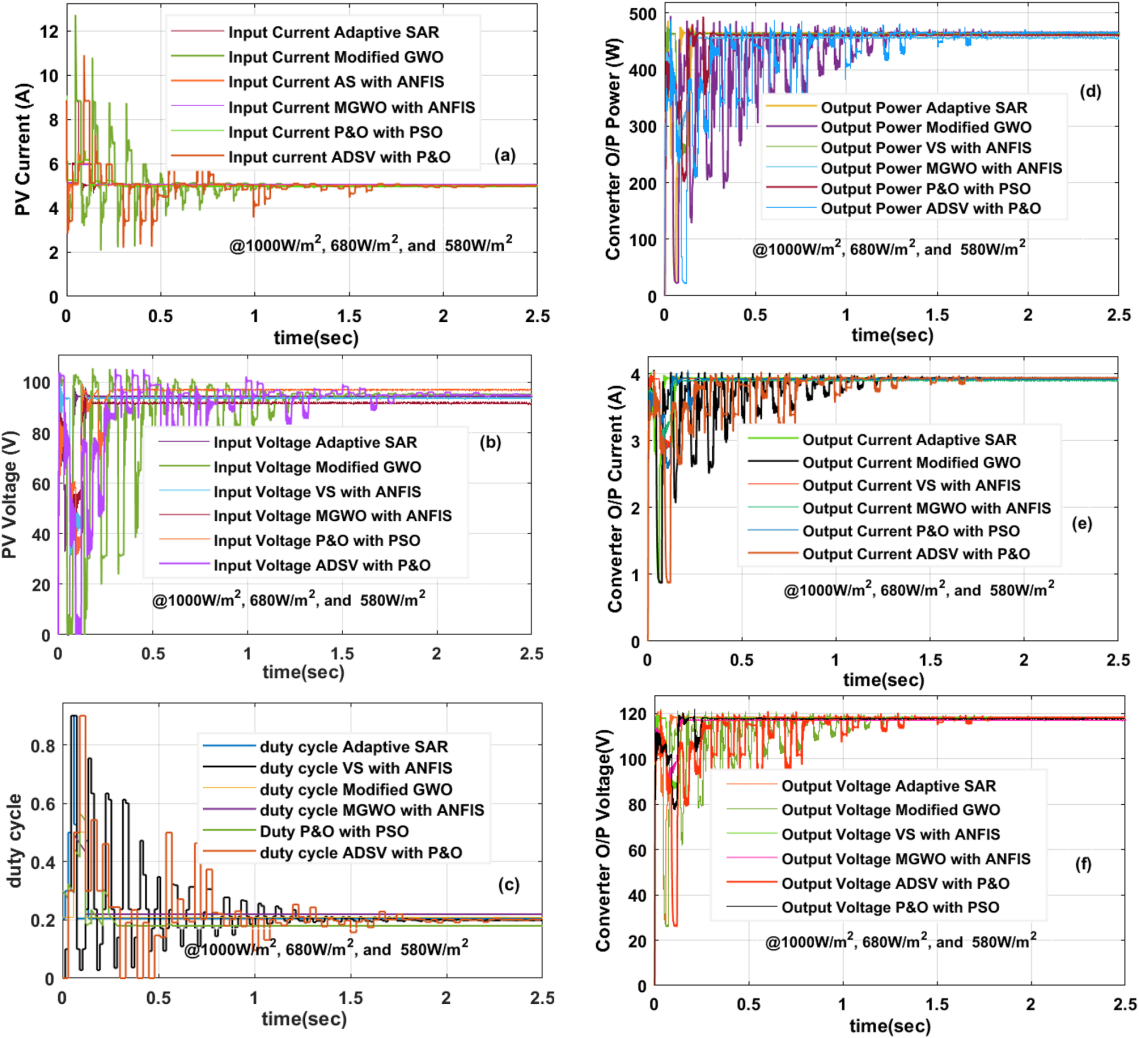
Under 1000W/m^2^, 680W/m^2^, and 580W/m^2^, (**a**) PV panel current, (**b**) PV panel voltage, (**c**) the duty cycle of the converter, (**d**) Power of converter, (**e**) the current of the converter, plus (**f**) voltage of converter.

Generally, all types of PV systems work at various sunlight thermal conditions. The sunlight power is directly related to the PV cell voltage loss. In this third PSC, the achieved power, current, and voltages of the PV from the utilization of MGWO-ANFIS, P&O-PSO, modified GWO, VS-ANFIS, adaptive SAR, plus ADSV with P&O are 473.40W, 5.459A, 86.72 V, 471.73W, 5.441A, 86.7 V, 471.45W, 5.438A, 86.68 V, 470.39W, 5.43A, 86.62 V, 470.15W, 5.429A, 86.60 V, 468.57W, 5.412A, plus 86.58 V. From these calculations, the modified GWO-optimized ANFIS network is giving superior performance which is discussed in Fig. 11a, b. From Fig. 11c, the converter needed a very less value of duty by applying a modified GWO controller when equalized with the SAR controller. Also, the design cost of GWO is moderate. The converter gives the voltage and current signals which are explained in Fig. 11d, e. The efficiencies of MGWO-ANFIS, P&O-PSO, modified GWO, VS-ANFIS, adaptive SAR, plus ADSV with P&O-based PV system is 97.22%, 97%, 96.92%, 96.11%, 96.02%, plus 95.5%. The converter power settling time by applying the MGWO-ANFIS, P&O-PSO, modified GWO, VS-ANFIS, adaptive SAR, plus ADSV with P&O is 0.031 s, 0.039 s, 0.092 s, 0.22 s, 0.28 s, plus 0.38 s. The overall waveforms of converter power are given in Fig. 11f. The design cost of ADSV is very low. However, it does not apply to these rapid variations of sunlight insulations. The entire PV system performance under quick variation of solar insolations is given in Table 3.

**Table 3:** Investigated parameters of different power point identifying methods at quick changes in atmospheric conditions.

MPPT	PV Current	PV Voltage	PV Power	Converter Current	Converter Voltage	Converter Power	Efficiency	Stabilizing period	Distortions	Complexity in Design
Cloudy Irradiation Condition, 1000W/m^2^, 880W/m^2^, and 780W/m^2^										
ADSV with P&O	6.80A	86.13 V	585.68W	4.209A	134.00 V	564.00W	96.3%	0.15 s	High	Less
Adaptive SAR	6.82A	87.92 V	599.61W	4.310A	134.92 V	581.62W	97.0%	0.13 s	High	Less
VS with ANFIS	6.87A	88.62 V	608.81W	4.382A	135.00 V	591.58W	97.17%	0.1 s	Moderate	Moderate
Modified GWO	6.88A	88.75 V	610.6W	4.3649A	136.22 V	594.60W	97.38%	0.08 s	Moderate	Moderate
P&O with PSO	6.90A	89.0 V	614.10W	4.343A	138.41 V	601.14W	97.89%	0.028 s	Low	High
MGWO-ANFIS	6.92A	89.2 V	617.26W	4.259A	142.32 V	606.15W	98.20%	0.02 s	Low	Moderate
Cloudy Irradiation Condition, 1000W/m^2^, 780W/m^2^, and 680W/m^2^										
ADSV with P&O	6.17A	86.00 V	530.62W	4.007A	127.11 V	509.39W	96.00%	0.25 s	High	Less
Adaptive SAR	6.18A	87.18 V	538.77W	4.086A	127.59 V	521.42W	96.78%	0.21 s	High	Less
VS with ANFIS	6.2A	87.22 V	540.76W	4.100A	127.66 V	523.50W	96.81%	0.18 s	Moderate	Moderate
Modified GWO	6.212A	87.39 V	542.86W	4.128A	127.81 V	527.60W	97.19%	0.098 s	Moderate	Moderate
P&O with PSO	6.25A	87.40 V	546.25W	4.156A	128.12 V	532.59W	97.5%	0.034 s	Low	High
MGWO-ANFIS	6.29A	87.17 V	548.29W	4.714A	128.71 V	537.32W	98.00%	0.027 s	Low	Moderate
Cloudy Irradiation Condition, 1000W/m^2^, 680W/m^2^, and 580W/m^2^										
ADSV with P&O	5.412A	86.58 V	468.57W	3.825A	116.97 V	447.48W	95.50%	0.3 s	High	Less
Adaptive SAR	5.429A	86.60 V	470.15W	3.855A	117.10 V	451.43W	96.02%	0.28 s	High	Less
VS with ANFIS	5.43A	86.62 V	470.39W	3.8511A	117.39 V	452.09W	96.11%	0.22 s	Moderate	Moderate
Modified GWO	5.38A	86.68 V	471.45W	3.865A	118.21 V	456.92W	96.92%	0.09 s	Moderate	Moderate
P&O with PSO	5.441A	86.7 V	471.73W	3.858A	118.59 V	457.57W	97.00%	0.039 s	Low	High
MGWO-ANFIS	5.459A	86.72 V	473.40W	3.841A	119.82 V	460.23W	97.22%	0.031 s	Low	Moderate

## Conclusion

The solar PV system investigation has been done by using the MATLAB Simulink tool at various sunlight shading conditions. Here, the 3-diode model PV cell is selected because of its accurate behavior under various PSCs. The 3-diode model PV array current versus voltage curve is nonlinear fashion. The nonlinear characteristics issue of PV is handled by using the MPPT controller. From the above simulation analysis, the modified grey wolf controller fed ANFIS technique is giving high peak power of PV, good dynamic response, less steady state distortions, required a smaller number of iterations, few sensors used for sensing the PV parameters, less design cost, easy to implement, more efficient, plus highly suitable for the cloudy conditions of the PV systems. Also, the PV power production cost is quite high value which is not a desirable feature for the present solar PV systems. The non-isolated DC-DC converter is connected to the output of the PV to improve the voltage profile of the supply. The merits of the utilized converter are easy understanding, high reliability, plus lower price to implement.

## Data Availability

The data used to support the findings of this study are included in the article.
